# PET/CT incidental detection of second tumor in patients investigated for pancreatic neoplasms

**DOI:** 10.1186/s12885-018-4469-4

**Published:** 2018-05-04

**Authors:** Lucia Moletta, Sergio Bissoli, Alberto Fantin, Nicola Passuello, Michele Valmasoni, Cosimo Sperti

**Affiliations:** 1 Department of Surgery, Oncology and Gastroenterology, 3rd Surgical Clinic, University of Padua, Padua, Italy; 2 Department of Nuclear Medicine, Castelfranco Veneto General Hospital, Castelfranco Veneto, Treviso, Italy; 3 Gastroenterology Unit, University of Padua, Padua, Italy

**Keywords:** Incidentaloma, Pancreas, Pancreatic neoplasms, Positron emission tomography, Surveillance

## Abstract

**Background:**

Positron Emission Tomography/computed tomography (PET/CT) is an imaging technique which has a role in the detection and staging malignancies (both in first diagnosis and follow-up). The finding of an unexpected region of FDG (Fluorodeoxyglucose) uptake can occur when performing whole-body FDG-PET, raising the possibility of a second primary tumor. The aim of this study was to evaluate our experience of second primary cancer incidentally discovered during PET/CT examination performed for pancreatic diseases, during the initial work-up or follow-up after surgical resection.

**Methods:**

In this study, a retrospective evaluation of a prospectively collected data base was performed. Three hundred ninety- nine patients with pancreatic pathology were evaluated by whole body PET/CT imaging from January 2004 to December 2014. Among them, 348 patients were scanned before surgical resection and 51 during the course of their follow-up (pancreatic cancer). Median follow-up time was 29 months (range 14-124).

**Results:**

Fifty-six patients (14%) had incidental uptake of FDG in their organs: 31 patients had focal uptake and 25 showed diffuse with or without focal uptake. All patients with focal uptake were investigated, and invasive malignancy was diagnosed in 22 patients: 14 colon, 4 lung, 1 larynx, 1 urothelial, 1 breast cancer, and 1 colon metastasis from pancreatic cancer. Twenty patients underwent resection, and 6 endoscopic removal of colonic polyps. Three patients were not operated for advanced disease, and two patients did not show any pathology (PET/CT false positive). Of the 10 patients investigated for diffuse uptake, no malignancy was found; none of these patients developed a second cancer during the follow-up.

**Conclusions:**

As in other malignancies, unexpected FDG uptake can occur in patients having PET/CT investigation for pancreatic diseases. Focal uptake is likely to be a malignancy and deserves further investigations, although the stage and the poor prognosis of primary pancreatic cancer should be kept in mind. Some selected patients may benefit from the aggressive treatment of incidental lesions and show survival benefit.

## Background

Positron Emission Tomography/computed tomography (PET/CT) is an imaging technique which has a role in the detection of malignancies and in cancer staging (both in first diagnosis and follow-up) [1, 2], including pancreatic neoplasms [3]. The finding of an unexpected region of FDG uptake can occur when performing whole-body FDG-PET; this raises the possibility of a second primary tumor [4]. In the past decade, incidental cancer has been detected by PET/CT in asymptomatic patients [5], patients with head neck cancer [6], oesophagogastric malignancies [7], lymphoma [8], and lung cancer [9]. Informations regarding PET/CT incidental cancer in patients with pancreatic neoplasms are still lacking. Aim of this study was to assess the frequency and significance of incidental findings reported by PET/CT scans in patients investigated for pancreatic lesions, both benign and malignant.

## Methods

Patients who underwent PET/CT examination for staging or follow-up after resection of pancreatic tumors observed in our Department from January 2004 to December 2014, were identified from a prospectively collected database. The scans were reviewed and any incidental findings recorded. One person (SB) assessed all PET/CT reports. An incidental finding was defined as a significant area of FDG uptake in a site unlikely to be related to the pancreatic neoplasm. FDG uptake was described according to focal or diffuse pattern assessed by the nuclear medicine physician. Clinical, radiographic, endoscopic, surgical and pathological records, and follow-up imaging were used as evaluation of incidental PET findings. A validation of the diagnosis was based on the pathologic findings of a resected specimen, biopsy examination, or follow-up. When an extrapancreatic or focal uptake of 18-FDG was detected, an additional diagnostic work-up was performed. All suspected lesions underwent histological confirmation. 18F-FDG PET scans were performed using standard clinical protocols by a hybrid system (Biograph Sensation 16, Siemens, provided with a multislice CT and LSO crystals). A minimum fasting time of 6 h was prescribed and blood glucose levels less than 120 mg/dl before intravenous injection of a weight-based amount of 18-FDG (37 MBq/10 kg) were obtained. The CT scans were performed without oral and/or intravenous contrast medium. Cross-sections for attenuation correction of the emission images (when the PET tomograph without CT was used) were obtained with two transmission scans of the abdomen by 68 Ge/68 Ga rod sources before the administration of 18-FDG. When using the hybrid system, CT scan was done with a kCp of 120 and weight-based amperage of 120 mAs. PET scan (for both tomographs) began 90 min after the tracer’s injection acquiring 2 beds (16 cm each) to cover upper abdomen area. Coronal, sagittal and transaxial sections were obtained for visual analysis. In the suspected neoplastic areas, a quantitative analysis was performed through the calculation of the maximum standardized uptake value (SUVmax, SUV = tissue tracer concentration/injected dose/body weight) which was analyzed by placing a circular region of interest (on transaxial sections) over the area of maximal focal 18-FDG uptake. We established a SUVmax cut-off of 2.5 or greater according to our previous experience. The PET scan was interpreted by a single observer (S.B.) without knowledge of the CT scan results.

## Results

**Fig. 1:**
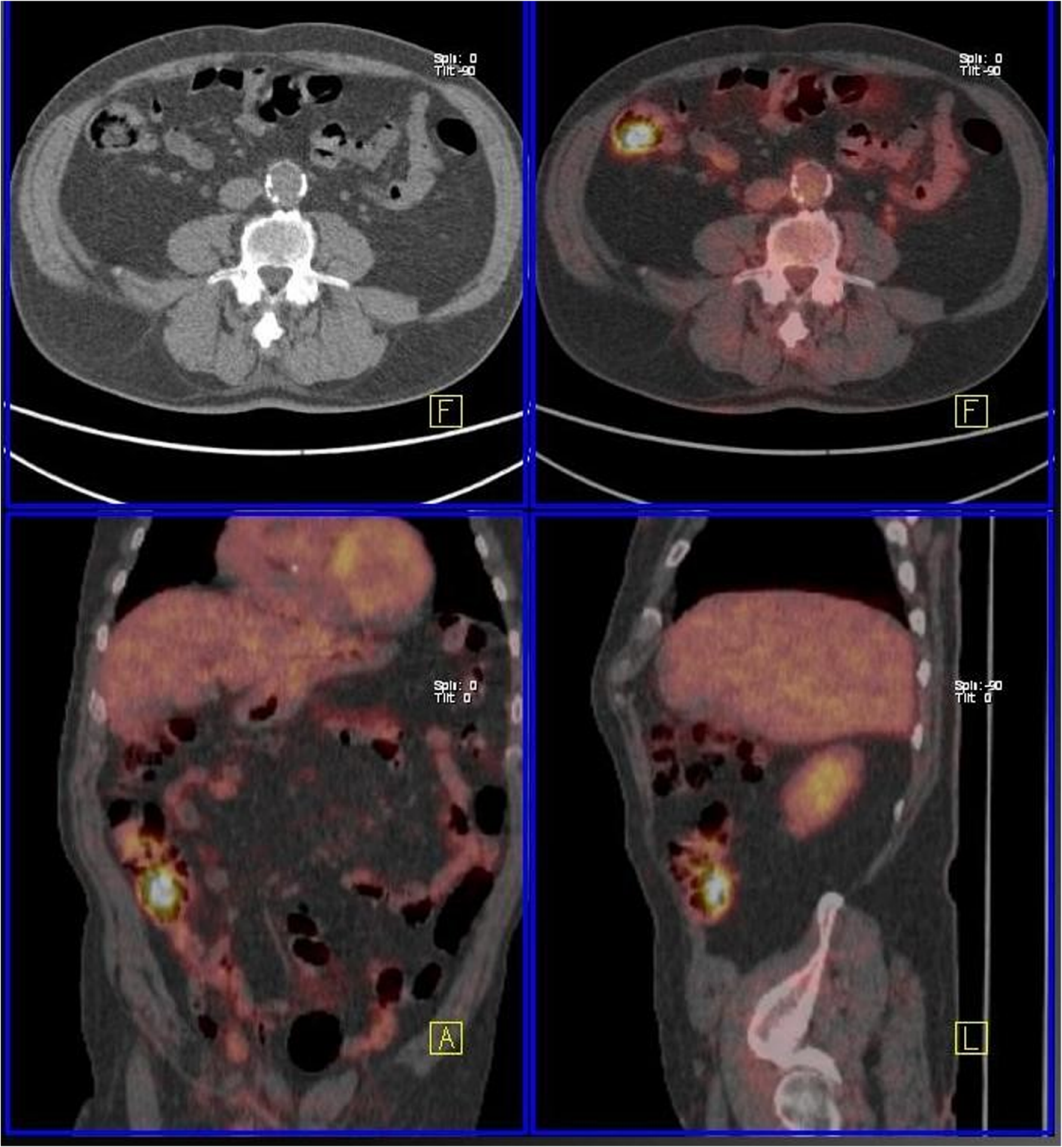
PET/CT incidental detection of cancer of the ascending colon associated with branch-type IPMN of the pancreatic head (PET negative)

**Fig. 2:**
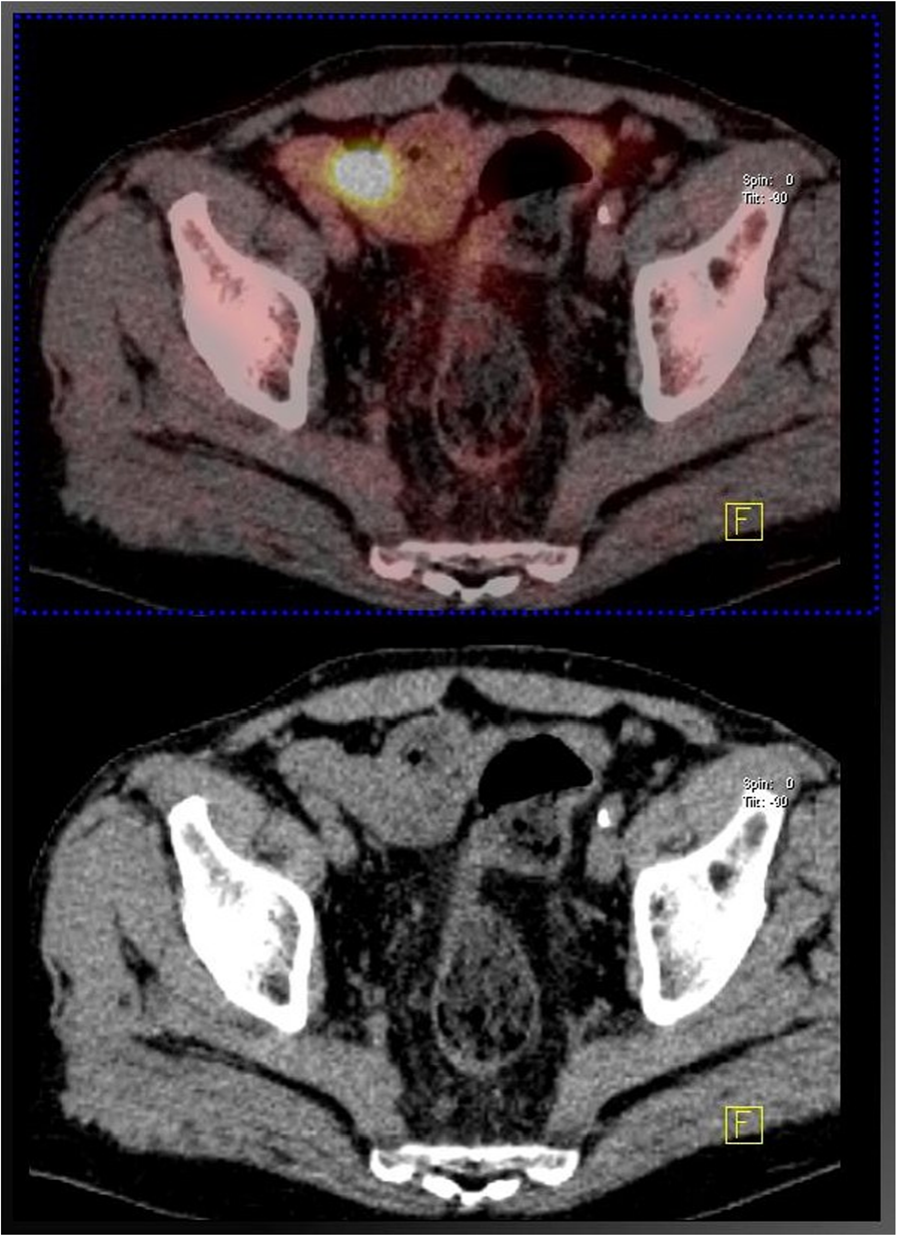
PET/CT incidental detection of cancer of the sigmoid colon 39 months after pancreatico-duodenectomy for pancreatic cancer

A total of 399 patients underwent PET/CT imaging during the study period: 348 in the initial work-up for pancreatic neoplasm, and 51 in the course of follow-up after resection for pancreatic cancer. Incidental FDG uptake was identified in 56 patients (14%): in these patients, pancreatic neoplasms included ductal adenocarcinoma (*n* = 32), intraductal papillary mucinous neoplasms (IPMNs, *n* = 21), cystic neoplasms (two mucinous tumors and 1 solid-pseudopapillary tumor). Forty-five patients underwent PET/CT at initial stage and 11 during the course of their follow-up after resection of pancreatic cancer. Thirty-one patients (55%) had focal uptake, while 25 (45%) showed diffuse with or without focal uptake. Focal uptake was observed in the colon (*n* = 21), lung (*n* = 4), rectum (*n* = 2), larynx (*n* = 1), urether (*n* = 1), breast (*n* = 1), duodenum (*n* = 1) (Table 1). Diffuse with or without focal uptake was observed in the colon (*n* = 20), rectum (*n* = 2), thyroid (*n* = 2), oesophagus (*n* = 1), stomach (*n* = 1) (Table 2). The colon was the most common site of FDG uptake in all groups (Figs. 1, 2). All patients with focal uptake were investigated with colonoscopy (*n* = 23), contrast-enhanced CT (*n* = 5), gastroscopy (*n* = 1), mammography (*n* = 1), laryngoscopy (*n* = 1). Investigations were performed in 10 patients with diffuse uptake (5 colonoscopy, 2 gastroscopy, 2 ultrasonography and fine-needle aspiration, 1 sigmoidoscopy).

**Table 1:** Site of incidental findings, results of investigation, treatment and outcome of patients with PET/CT extra-pancreatic focal uptake of the radiotracer

Site	Total Number of Patients	Pathology	Number of patients	Treatment	Outcome	
					N°	A / D *(mo)*
Colon	19	Adenocarcinoma	14	Colectomy	13	D
					1	A (61)
		Pancreatic cancer metastasis	1	CT	1	D (8)
		HGD	4	ER	4	D
Rectum	2	HGD	2	ER	1	D
					1	A (74)
Lung	4	Adenocarcinoma	3	Lobectomy	3	D
			1	CT	1	D (13)
Breast	1	Ductal Carcinoma	1	CT	1	D (7)
Ureter	1	Urothelial Cancer	1	Nephrectomy	1	D (21)
Duodenum	1	HGD	1	PD	1	A (72)
Larynx	1	Laryngeal Cancer	1	Laryngectomy	1	D (27)

*D* dead, *A* alive, *CT* chemotherapy, *ER* endoscopic resection, *PD* pancreaticoduodenectomy, *HGD* high grade dysplasia

**Table 2:** Site of incidental findings, results of investigation, reasons of non investigation of patients with pancreatic tumors who underwent PET/CT with diffuse uptake of the radiotracer

Site	Total Number of Patients	Investigated		Reason of Non Investigation	
		N°	Pathology	N°	Reason
Oesophagus	1	1	Oesophagitis	–	–
Stomach	1	1	Gastritis	–	–
Colon	20	5	1 Hyperplastic Polyps	8	Physiological uptake
				7	Advanced Disease
Rectum	2	1	Proctitis	–	–
Thyroid	2	2	Benign Goiter	–	–

**Fig. 3:**
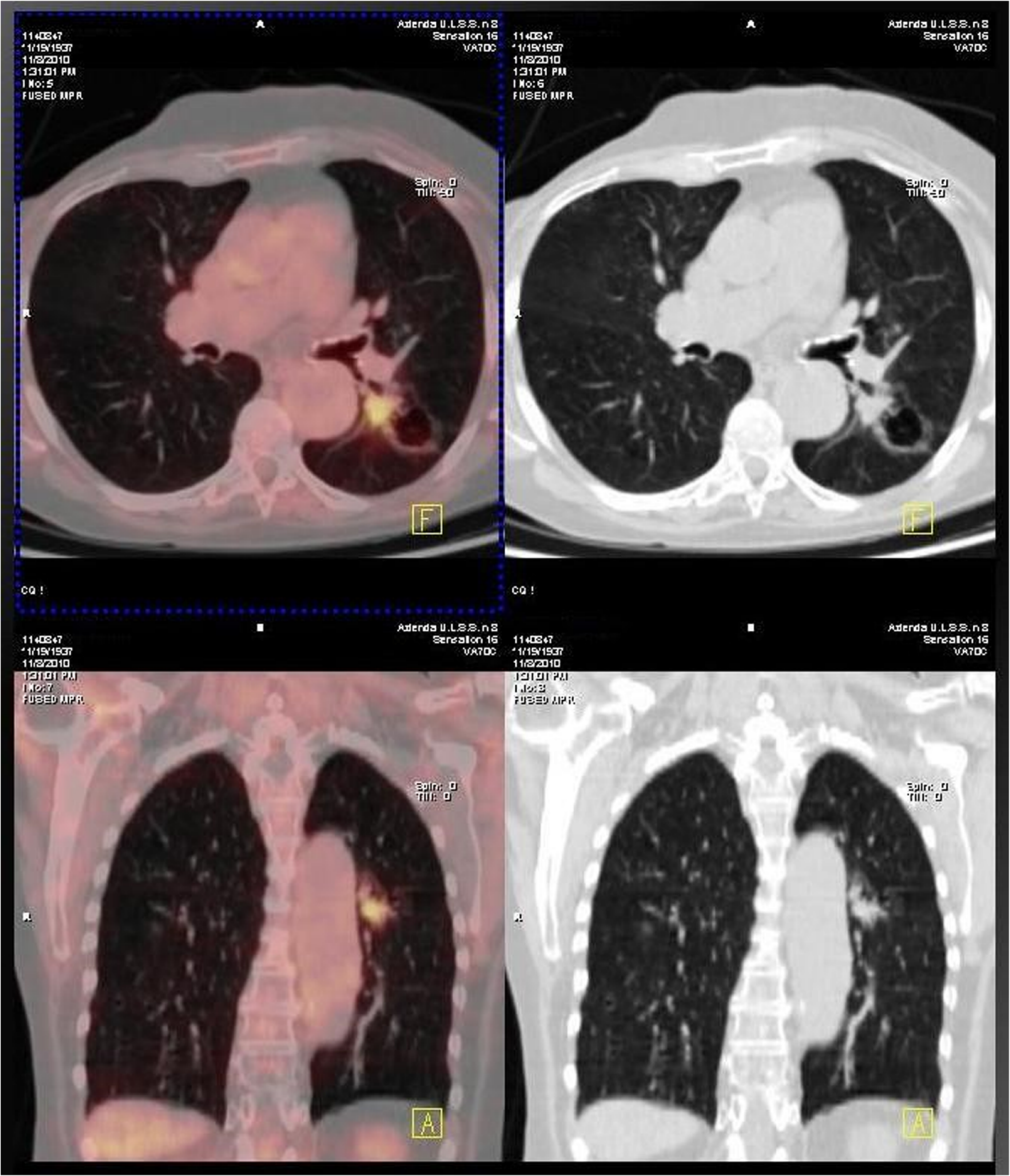
PET/CT incidental detection of left pulmonary adenocarcinoma 31 months after distal pancreatectomy for pancreatic cancer

**Fig. 4:**
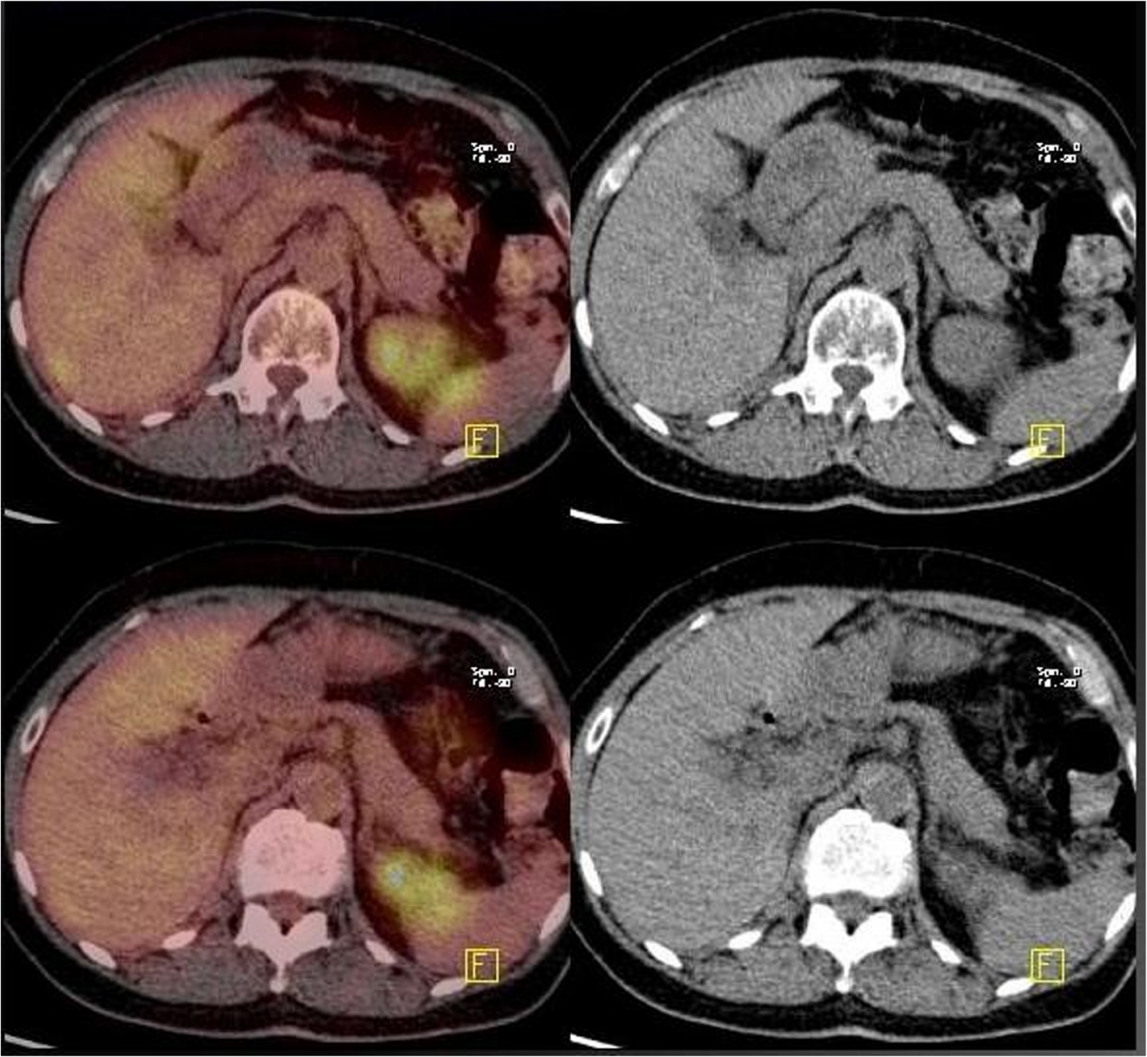
PET/CT incidental detection of left ureteral cancer associated with IPMN (adenoma) of the pancreatic tail (PET negative)

Of the 31 patients investigated for focal FDG uptake (19 patients with primary malignant tumors and 12 benign pancreatic neoplasms), invasive malignancy was diagnosed in 22 patients: 14 colon adenocarcinoma, 4 lung adenocarcinoma (Fig. 3), 1 cancer of the larynx, 1 urothelial cancer (Fig. 4), 1 breast cancer, and 1 colon metastasis from pancreatic cancer. Among the remaining 9 patients, 7 showed colonic (*n* = 6) or duodenal (*n* = 1) polyps with high-grade dysplasia, and two patients did not show any pathology. Fourteen patients underwent colectomy, 6 endoscopic removal of colonic polyps, 3 lung lobectomy, 1 laryngectomy, 1 pancreaticoduodenectomy, and 1 nephrectomy. Five patients did not undergo surgery: three for advanced disease (1 lung cancer, 1 breast cancer, and 1 pancreatic cancer metastasis), and two for not evidence of disease (false positive). Among patients with pathological uptake of FDG during initial work-up for pancreatic cancer (*n* = 7 patients), in 5 patients detection of second tumor changed the operative management and a colectomy was associated with the pancreatic resection. The other two patients did not undergo pancreatic resection for advanced cancer. After a median follow-up of 29 months (range 14-96 months), 2 patients investigated for pancreatic cancer are alive and free of disease, 4 and 5 years after resection of lung cancer and colon cancer, respectively. The remaining patients died of progression of disease, with a median overall survival of 20 months (range 5-96 months), with 5 long survivors (survival > 60 months) .

Among the 12 patients investigated for benign pancreatic disease (9 IPMNs and 3 cystadenomas), 8 patients are alive and disease-free (median survival time 48 months) while 2 patients died for colon cancer (*n* = 1) and urothelial cancer (*n* = 1) 37 and 16 months after surgery, respectively. The remaining 2 patients died for causes unrelated to cancer.

Of the 10 patients investigated for diffuse uptake, hyperplastic colonic polyps were detected in 1 patient, proctitis in 1, gastritis in 1, oesophagitis in 1, thyroid goiter in 2, no abnormality in the remaining 4 patients (Table 2). None of these patients developed a second cancer during the entire follow-up. Quantitative analysis did not show significant difference between lesions with focal uptake (mean SUV = 6.4, range 3.0-12.5) and diffuse uptake (mean SUV 5.1, range 2.5-10.0).

A total of 51 patients underwent PET/CT during the follow-up after resection of pancreatic cancer. Eleven patients showed extrapancreatic focal uptake of FDG and 9 had resection of confirmed second cancer; one patient is alive 61 months after operation, while 8 patients died for recurrent pancreatic or colon cancer with a median survival time of 41 months (range 13-118 months). The remaining two patients did not have evidence of disease (colon) and are still alive and well after 1 and 3 years, respectively (Table 3).

**Table 3:** Site of incidental findings, results of investigation of patients who underwent PET/CT with focal uptake of the radiotracer during the follow-up after resection of pancreatic tumors

Site	Total Number of Patients	Investigated	
		N°	Pathology
Lung	2	2	Cancer
Larynx	1	1	Cancer
Colon	7	7	5 Cancer + 2 FP^a^
Rectum	1	1	HGD polyp

*FP* false positive, *HGD* high grade dysplasia

^a^ two patients with colonic focal uptake were investigated with colonoscopy and CT scan, which resulted negative for pathology in both cases

## Discussion

FDG-PET offers the unique opportunity to provide not only whole-body images, but also metabolic or functional informations regarding the tumor tissue. Moreover, the widespread availability of this imaging technique led to increasing number of incidental findings: this necessarily suggest further investigations in order to exclude malignancies. As previously reported by other studies dealing with PET/CT in different malignancies, we have observed an increasing number of extrapancreatic FDG uptake in patients with pancreatic neoplasms. So, we evaluated our experience of incidental findings of PET/CT performed for patients with neoplasms of the pancreas: to our knowledge this is the first study dedicated to this specific topic. An incidental finding was detected in 14% of the patients in our study, and it was further investigated in 72% of cases (all with focal and 10 out of 25 patients with diffuse uptake). Second malignant or premalignant lesions were confirmed in all but one patient who showed PET/CT with focal uptake: the remaining patient with a focal FDG uptake had a colonic metastasis from pancreatic cancer confirmed by pathologic examination. The number of incidental findings in our study appears higher than that reported by other authors [10, 11] but much greater incidence (more than 20%) has been reported for other types of primary neoplasms [9, 12].

Most of our PET/CT incidental findings were localized in the large bowel, in according with previous studies including patients with different primary tumors [13–15]. In our study a malignant or premalignant lesion was detected in 74% of patients who underwent endoscopy. A confirmed pathologic lesion was obtained in all but two patients with focal FDG uptake, while none of the patients investigated for diffuse FDG uptake showed unexpected tumors. This finding outlines the concept that when a focal uptake of FDG is detected, a high suspicion of malignancy is suggested, and further investigations are necessary [16–18]. One could argue that detection of a second tumor in patients with such an aggressive disease as pancreatic cancer does not modify the poor prognosis, and it is without a clinical significance. However, in our experience, some patients who underwent resection of a second neoplasm incidentally detected by PET/CT, showed prolonged survival: this is particularly true for lesions incidentally detected in the follow-up of patients without recurrence after resection of pancreatic cancer. So, as we previously reported [19], PET/CT is useful not only for the staging of pancreatic malignancy, but also for the postoperative surveillance of resected pancreatic cancer. Moreover, it has been reported that IPMNs are at risk of association with extra-pancreatic malignancies [20, 21], but contrasting results are also suggested [22, 23]. In our study, 21 patients with benign IPMNs showed extra-pancreatic PET/CT incidental findings: in 11 patients a focal uptake of the radiotracer was evident, and a second malignancy was resected in 9.

In our Center, after an initial period when PET/CT was performed (whenever possible) in almost all patients with pancreatic cancer, we now perform it to stage resectable cancer and, when indicated, during the follow-up of resected patients. Moreover, we think that PET/CT can be an useful tool also during follow-up of selected patients with IPMNs. On the contrary, we have observed that PET/CT is unlikely to modify the management of patients with metastatic pancreatic cancer.

## Conclusions

As in other tumors, our study reveals that unexpected FDG uptake is frequent in patients having PET/CT investigation for pancreatic neoplasms. Focal uptake is likely to be a malignancy and deserves accurate examination, although the stage and the poor prognosis of primary cancer should be kept in mind. Some selected patients (with benign IPMN, or in follow-up after resection of pancreatic cancer) may benefit from the aggressive treatment of incidental lesions and show survival benefit.

## Abbreviations

18F-FDG: 18F-Fluorodeoxyglucose
A: Alive
CT: Chemotherapy
D: Dead
ER: Endoscopic resection
FP: False positive
HGD: High grade dysplasia
IPMN: Intraductal papillary mucinous neoplasms
PD: Pancreaticoduodenectomy
PET/CT: Positron emission tomography/computed tomography
SUV: Standardized uptake value
